# Gas‐Sensitive Cellulosic Triboelectric Materials for Self‐Powered Ammonia Sensing

**DOI:** 10.1002/advs.202203428

**Published:** 2022-08-26

**Authors:** Wanglin Zhang, Jiamin Zhao, Chenchen Cai, Ying Qin, Xiangjiang Meng, Yanhua Liu, Shuangxi Nie

**Affiliations:** ^1^ School of Light Industry and Food Engineering Guangxi University Nanning 530004 P. R. China

**Keywords:** cellulose nanofibril, gas‐sensitive triboelectric materials, self‐powered sensing, triboelectric nanogenerators, wireless sensing

## Abstract

Gas‐sensitive materials are capable of dynamic identification and content monitoring of specific gases in the environment, and their applications in the field of gas sensing are promising. However, weak adsorption properties are the main challenge limiting the application of gas‐sensitive materials. A highly adsorbent gas‐sensitive cellulose nanofibril (CNF)‐based triboelectric material with a layered structure is prepared here and it is applied to self‐powered gas sensing. The layered structure of the triethoxy‐1H,1H,2H,2H‐tridecafluoro‐n‐octylsilane cellulose nanofiber (PFOTES‐CNF)‐based gas‐sensitive material further enhances the adsorption of the material due to electrostatic adsorption in the electrostatic field induced by triboelectricity. It is found that the ammonia‐sensitive material obtained by loading Ti_3_C_2_T*_x_* in PFOTES‐CNF has a fast response/recovery (12/14 s), high sensitivity response (*V*_air_/*V*_gas_ = 2.1), high selectivity response (37.6%), and low detection limit (10 ppm) for 100 ppm of ammonia gas. In addition, the ammonia‐sensitive CNF‐based triboelectric material can accurately identify NH_3_ concentration changes in the range of 10–120 ppm and transmit the signal wirelessly to the user interface, facilitating real‐time online monitoring of NH_3_ in the environment. A novel strategy is provided here for designing and preparing high‐performance gas‐sensitive composites and the analysis of self‐powered gas sensing is guided.

## Graphical abstract

A gas‐sensitive cellulose nanofiber (CNF)‐based triboelectric material with a layered structure is prepared here and it is applied to self‐powered gas sensing. The ammonia‐sensitive material obtained by loading Ti_3_C_2_T*_x_* in triethoxy‐1H,1H,2H,2H‐tridecafluoro‐n‐octylsilane‐cellulose nanofibril (PFOTES‐CNF) has a fast response/recovery (12/14 s), high sensitivity response (*V*_air_ */V*_gas_ = 2.1), high selectivity response (37.6%), and low detection limit (10 ppm) for 100 ppm of ammonia gas.

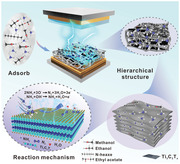

## 1 Introduction

Gas‐sensitive materials are new functional materials that can reflect the composition or concentration of a specific gas in the environment promptly. It is widely popular because of its simple production process and low cost.^[^ ^1^ ^]^ Thanks to these unique advantages, it has been commonly used in the field of gas sensing in recent years. The most common gas‐sensitive materials are mainly inorganic semiconductors WO_3_,^[^ ^2^ ^]^ ZnO,^[^ ^3^ ^]^ In_2_O_3_,^[^ ^4^ ^]^ SnO_2_,^[^ ^5^ ^]^ etc. The physical or chemical properties of these materials change significantly when exposed to specific gases. Based on these changes, the specific gas compositions or concentrations are identified by researchers.^[^ ^6^ ^]^ Although these gas‐sensitive materials can sense gases at certain concentrations, some pressing issues remain, such as weak adsorption capacity.^[^ ^7^ ^]^ Therefore, developing a gas‐sensitive material with high adsorption capacity is highly anticipated.

In contrast, cellulose has a neat 1D layered structure and is rich in oxygen‐containing polar functional groups (e.g., hydroxyl groups),^[^ ^8^ ^]^ which will lead to less insulating contacts between 2D conductive nanosheets.^[^ ^9^ ^]^ The formation of reactive end groups (such as F, O, and OH) on the surface of Ti_3_C_2_T*_x_* sheets allows cellulose nanofibers (CNFs) to be easily adsorbed on the surface of Ti_3_C_2_T*_x_* nanosheets. The 2D structure of Ti_3_C_2_T*_x_* nanosheets and the 1D structure of CNFs are often used as “bricks” and “mortar” to form multilayer structures.^[^ ^10^ ^]^ The layered structure gives the material a high specific surface area, facilitating electrostatic adsorption between gas molecules and cellulose chains and surface catalytic reactions.^[^ ^11^ ^]^ Meanwhile, the abundant hydroxyl groups on the molecular chain provide considerable modifiability and facilitate the introduction of specific gas‐sensitive materials. For example, Ti_3_C_2_T*_x_*, a novel 2D carbide with large termination groups on the surface, has good selectivity for gases at room temperature and is commonly used as a specific gas‐sensitive material.^[^ ^12^ ^]^ It provides a new solution for developing highly adsorptive, gas‐sensitive materials.

Recently, triboelectric nanogenerators (TENG), a novel sustainable and green energy harvesting device,^[^ ^13^ ^]^ can convert external mechanical stimuli into electrical signals, allowing them to be used as pressure,^[^ ^14^ ^]^ tactile,^[^ ^15^ ^]^ or self‐powered gas sensors.^[^ ^6^, ^16^ ^]^ Cai et al. constructed a contact‐separated TENG as self‐powered ammonia (NH_3_) sensor using carbon nanotube materials and fluorinated ethylene propylene to achieve real‐time monitoring of NH_3_.^[^ ^17^ ^]^ Cui et al. designed a novel self‐powered gas sensor using polyaniline nanofibers as triboelectric electrodes in TENG.^[^ ^7^ ^]^ Zhang et al. demonstrated that the Berlin green framework is a promising sensing material for ammonia detection by density generalized theory simulations and experimental gas sensing studies.^[^ ^18^ ^]^ A self‐powered complete ammonia leak monitoring device was demonstrated by Feng et al.^[^ ^19^ ^]^ Yu et al. prepared an organ‐like Ti_3_C_2_T*_x_* MXene/metal‐organic framework‐derived copper oxide (CuO) gas sensor.^[^ ^20^ ^]^ These efforts achieve self‐powered sustainable monitoring of the gas, effectively alleviating the problem of high energy consumption for the work of gas‐sensitive materials. Notably, CNF contains abundant hydroxyl groups with a considerable chemical modifying properties and has a wide range of applications in TENG.^[^ ^21^ ^]^ Therefore, developing a CNF‐based gas‐sensitive triboelectric material for self‐powered gas sensing is a promising approach to developing gas‐sensitive materials with high adsorption capacity, which is of great importance for gas‐sensitive material applications.

Herein, this work develops gas‐sensitive CNF triboelectric materials with a layered structure to provide sustainable output for real‐time self‐powered gas sensing. The layered structure of triethoxy‐1H,1H,2H,2H‐tridecafluoro‐n‐octylsilane cellulose nanofibril (PFOTES‐CNF)‐based gas‐sensitive material has excellent chemical stability and high specific surface area, which further enhances the gas adsorption of the material due to electrostatic adsorption in the electrostatic field induced by triboelectric electricity. Loading Ti_3_C_2_T*_x_* in PFOTES‐CNF resulted in an ammonia‐sensitive material with a selective response of 37.6% to 100 ppm ammonia (2.5 times higher than the second‐highest response gas), a response recovery time (12/14 s), and a low monitoring limit (10 ppm). In addition, the ammonia‐sensitive CNF‐based triboelectric material can accurately identify NH_3_ concentration changes in the range of 10–120 ppm and transmit the signal wirelessly to the user interface, facilitating real‐time online monitoring of NH_3_ in the environment. This study provides a novel strategy for developing and utilizing high‐performance gas‐sensitive materials and offers promising applications in the field of self‐powered wireless gas sensing.

## 2 Results and Discussion

### 2.1 Cellulosic Triboelectric Materials for Self‐Powered Gas Sensing

**Figure 1:**
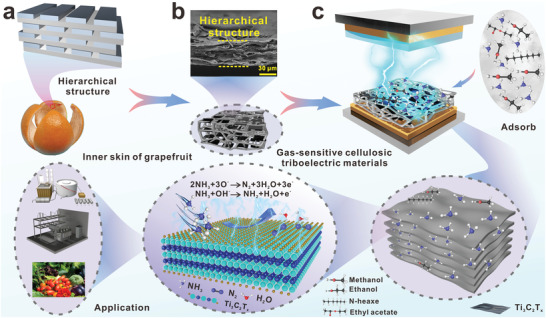
Cellulosic triboelectric materials for self‐powered gas sensing. a) Laminar structure of the inner surface of grapefruit peel. b) Gas‐sensitive CNF base triboelectric material cross‐section layered structure. c) Schematic diagram of gas‐sensitive CNF‐based triboelectric material for self‐powered gas sensing.

Over time, biological tissue has evolved to develop unique functional structures with excellent physicochemical properties.^[^ ^22^ ^]^ For example, the laminar structure inside the grapefruit peel effectively enhances gas absorption (**Figure** **1**a).^[^ ^23^ ^]^ The hierarchical structure of biomaterials dramatically increases the surface area, depending on the number of interfaces between adjacent layers and the surface area.^[^ ^24^ ^]^ Based on the above understanding, this paper chose to modify the cellulose by triethoxy‐1H,1H,2H,2H‐tridecafluoro‐*n*‐octylsilane (PFOTES). It is then blended with Ti_3_C_2_T*_x_* by “intermittent filtration” to prepare a layered structure of ammonia‐sensitive CNF‐based triboelectric materials (Figure 1b). The Ti_3_C_2_T*_x_* sheets are arranged in layers under the string guidance of cellulose molecular chains, and the layered structure formed increases the surface area of cellulose and Ti_3_C_2_T*_x_* sheets.^[^ ^25^ ^]^ This ammonia‐sensitive triboelectric material was assembled into a TENG for self‐powered gas sensing application (Figure 1c). Compared with other volatile organic gases (VOCs), this ammonia‐sensitive material has superior adsorption of NH_3_. The NH_3_ molecule is monitored in real‐time by binding to the −OH, −O functional group on the surface of Ti_3_C_2_T*_x_*, which leads to a reduction in the microcapacitor network and a decrease in the output performance, and ultimately by the change in the output electrical performance data for NH_3_ (Figure 1c). Real‐time self‐powered monitoring will effectively assist in NH_3_ content monitoring in chemical plants, NH_3_ storage units, and food products and also offers considerable application prospects for self‐powered gas sensing.

**Figure 2:**
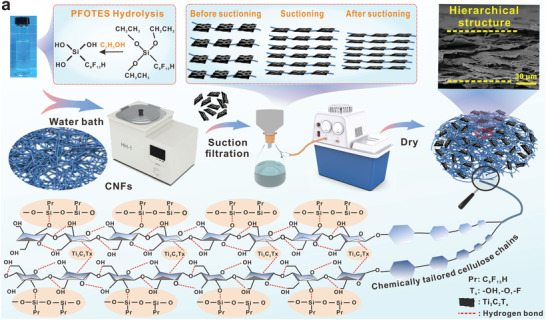
Preparation of gas‐sensitive cellulosic triboelectric materials. a) Preparation process of layered structured gas‐sensitive CNF‐based triboelectric materials.

This work utilizes intermittent pumping to construct layered structures of gas‐sensitive CNF‐based triboelectric materials and the method is simplified in terms of fabrication procedures. As shown in **Figure** **2**a, the nonhydrolyzed PFOTES is difficult to react with CNF. Therefore, water and a catalyst (usually an acid or a base) are first used to hydrolyze and release the silanol to generate the active hydroxyl group.^[^ ^26^ ^]^ Then it is mixed with CNF and water‐bathed, during which the hydrolyzed functional silanol monomer or oligomer is physically adsorbed onto the hydroxyl groups of CNFs. The introduction of F‐functional groups on the chains under the action of PFOTES will effectively improve the electron‐losing ability of CNF, which in turn will enhance the triboelectric properties.^[^ ^21b^ ^]^ Ti_3_C_2_T*_x_* was added to the CNF solution after a water bath and stirred. The functional groups such as −O and −OH in Ti_3_C_2_T*_x_* formed hydrogen bonds with the residual −OH on the CNF chains and arranged in layers under the string guidance of cellulose molecular chains. This is followed by intermittent filtration, where the filtrate is pumped in five stages to ensure retention of the layered structure. As the number of draws increases, the spacing between the sheets in the layered arrangement gradually becomes smaller, creating a layered structure progressively. Finally, the filter membrane is removed under a sheet former and compressed vertically to remove water and make a layered structure by adhesion between the CNF layers. The layered structure is observed in the cross‐section of the film, indicating the layered arrangement of CNF chains while maintaining their aligned laminar structure. In brief, a simple fabrication procedure successfully constructed gas‐sensitive CNF‐based triboelectric materials with layered structures.

### 2.2 Characterizations of the Gas‐Sensitive Cellulosic Triboelectric Materials

**Figure 3:**
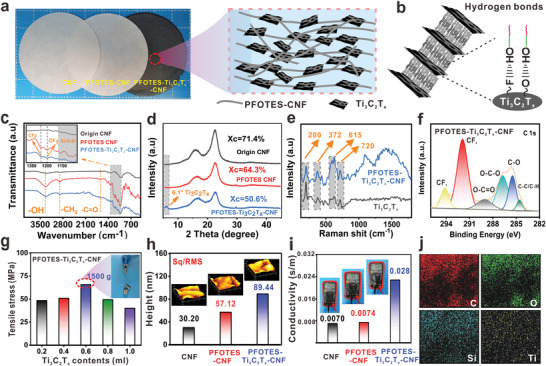
Characterization of gas‐sensitive cellulosic triboelectric materials. a) Physical diagram and internal structure of the three films. b) Schematic diagram of intrafilm cellulose connection with Ti_3_C_2_T*_x_*. c) FTIR spectra of films in the presence of different conditions. d) XRD spectra of films in the presence of different conditions. e) Raman spectra of gas‐sensitive cellulosic triboelectric materials. f) High‐resolution C1s peaks of gas‐sensitive cellulosic triboelectric materials. g) Comparison of mechanical properties of films with different Ti_3_C_2_T*_x_* content. h) Comparison of surface roughness of three types of films. i) Comparison of conductivity of three types of films. j) Mapping diagram of PFOTES‐Ti_3_C_2_T*_x_*‐CNF films.

The layered structure was formed by intermittent pumping, and the CNF film, PFOTES‐CNF film, and PFOTES‐Ti_3_C_2_T*_x_*‐CNF film were finally obtained by complete water removal after hot pressing drying in the sheet former (**Figure** **3**a). The −OH remaining on the cellulose macromolecule in the film is connected to the end functional groups of the sheet by hydrogen bonds and arranged in layers (Figure 3b). It is revealed by transmission electron microscope (TEM) images that Ti_3_C_2_T*_x_* flakes are delaminated and they exist as very thin 2D materials (Figure S1a, Supporting Information). Ti_3_C_2_T*_x_* flakes are almost transparent to electrons as the carbon lacey is seen. This strongly suggests a very thin film, considering the high atomic number of Ti (22) compare to the carbon (6).^[^ ^27^ ^]^ In pure Ti_3_C_2_T*_x_*, Figure S1b,c in the Supporting Information demonstrates the lattice fringe spacings were 0.242 nm corresponding to the (101) of Ti_3_C_2_T*_x_*. Moreover, Figure S1d in the Supporting Information demonstrates the stable presence of Ti_3_C_2_T*_x_*, while a little hydrolyzed residual PFOTES‐CNF is also present. Figure S1e in the Supporting Information demonstrates that the lattice fringe spacings were 0.246 nm corresponding to the (101) of Ti_3_C_2_T*_x_*. The lattice spacing parameter of Ti_3_C_2_T*_x_* in Figure S1f in the Supporting Information supports the structural retention of Ti_3_C_2_T*_x_* intact. Here, surface functional groups provide many defects that give more active states to gas adsorbing, and layered structure offered an efficient electron transport channel, both beneficial for gas detecting.^[^ ^28^ ^]^ Figure S1g,h in the Supporting Information shows the mapping results for Ti_3_C_2_T*_x_* and PFOTES‐Ti_3_C_2_T*_x_*‐CNF. The successful action of both Ti_3_C_2_T*_x_* and PFOTES is demonstrated by the characteristic elements Ti and Si. The surface functional groups of the films were characterized using Fourier transform infrared spectroscopy (FTIR) to evaluate the surface modification effect. Figure 3c shows the spectra of CNF at 4000–400 cm^−1^ before and after PFOTES modification and after the addition of Ti_3_C_2_T*_x_*. PFOTES‐CNF showed a new absorption peak at 1400–1000 cm^−1^ and two new absorption peaks at 1248 and 1183 cm^−1^. These correspond to the characteristic stretching vibrations of CF_2_ and CF_3_, respectively.^[^ ^26^ ^]^ In addition, the absorption peaks at 1100–1000 cm^−1^ belong to the typical peaks of Si—O—Si and Si—O—C.^[^ ^26^ ^]^ After adding Ti_3_C_2_T*_x_* again, the characteristic peak of Ti_3_C_2_T*_x_* appeared at 1387, which confirmed that CNF had been successfully modified by PFOTES and successfully complexed with Ti_3_C_2_T*_x_*. Ti_3_C_2_T*_x_* has two typical peaks at 1440 and 584 cm^−1^, corresponding to the surface terminal group of C−F and −OH, respectively. After being mixed with PFOTES‐CNF, the cellulose characteristic absorption bands at 2890 (C−H stretching), 1634 (−OH bending), and 691 cm^−1^ (−OH out‐of‐plane bending) are observed in the FTIR spectrum of the PFOTES‐Ti_3_C_2_T*_x_*‐CNF films (Figure S2, Supporting Information).^[^ ^10^ ^]^ The FTIR plots of the films with different Ti_3_C_2_T*_x_* contents are shown in Figure S3 in the Supporting Information. XRD further verified the successful preparation of PFOTES‐Ti_3_C_2_T*_x_*‐CNF films and the characteristic diffraction peak of Ti_3_C_2_T*_x_* appeared at 6.1° (Figure 3d). Compared with the pure CNF films, the crystallinity of the films showed a significant decrease after PFOTES modification and the addition of Ti_3_C_2_T*_x_*, which further demonstrated the successful binding of silanol monomer and Ti_3_C_2_T*_x_* to the hydroxyl groups on CNF. The X‐ray diffraction (XRD) patterns of the films with different Ti_3_C_2_T*_x_* contents are shown in Figure S4 in the Supporting Information. As shown in Figure 3e, the Ti_3_C_2_T*_x_* film exhibits the characteristic peaks at 200, 372, 615, and 720 cm^−1^, corresponding to the vibrations of the Ti—C bonds of the exfoliated Ti_3_C_2_T*_x_*.^[^ ^27^, ^29^ ^]^ Meanwhile, in the PFOTES‐Ti_3_C_2_T*_x_*‐CNF films, the characteristic peaks of Ti_3_C_2_T*_x_* were observed. This is also in agreement with the previously reported work.^[^ ^30^ ^]^ The fractional peaks located at 283.58, 284.80, 287.46, and 289.25 eV in the X‐ray photoelectron spectroscopy (XPS) (C1s) measured spectra (Figure 3f) correspond to C—C, C—O, O—C‐—O, and O—C=O, respectively, which remain consistent with the characteristic peaks of pure CNF. However, new peaks appear at 291.4 and 293.6 eV, corresponding to —CF_2_ and —CF_3_, which further successfully modified CNF.^[^ ^26^ ^]^ The full XPS spectra of the thin film, O1s, and Si1s regions are shown in Figure S5a–c in the Supporting Information.

The excellent alignment of Ti_3_C_2_T*_x_* flakes and the surface morphology of the final PFOTES‐Ti_3_C_2_T*_x_*‐CNF films are crucial for their mechanical properties, surface roughness, and electrical conductivity. Comparative tensile stresses of films with different Ti_3_C_2_T*_x_* contents are shown in Figure 3g. When the Ti_3_C_2_T*_x_* content was 0.6 vol%, the tensile stress reached the maximum (67 Mpa) and was able to lift a weight of 1500 g. The tensile stresses of the remaining Ti_3_C_2_T*_x_* content films were lower mainly. Too little or too much Ti_3_C_2_T*_x_* content will lead to incomplete formation of the layered structure, which will lead to self‐stacking of Ti_3_C_2_T*_x_* nanosheets and ultimately affect their mechanical properties. Figure 3h shows the roughness of the three films detected by atomic force microscopy (AFM). The surface Sq/Rms of the modified and Ti_3_C_2_T*_x_*‐doped films were 57.12 and 89.44 nm, and the surface Sq/Rms of the pure CNF films was 30.12 nm, confirming that the PFOTES modification and layered structure effectively improved the surface roughness. The conductivity of the three films obtained by the conductivity tester Figure 3i confirmed that the conductivity of the films did not change much before, and after the modification, the addition of Ti_3_C_2_T*_x_* enhanced the conductivity of the films, resulting in conductivity of about four times higher than that of the pure CNF films. In addition, the energy dispersive spectroscopy (EDS) mapping (Figure 3j) of PFOTES‐Ti_3_C_2_T*_x_*‐CNF films showed that C, O, Si, and Ti elements were uniformly distributed, which indicated that silanol monomer and Ti_3_C_2_T*_x_* could be stably present in the films.

### 2.3 Triboelectric Properties of the Cellulosic Materials

**Figure 4:**
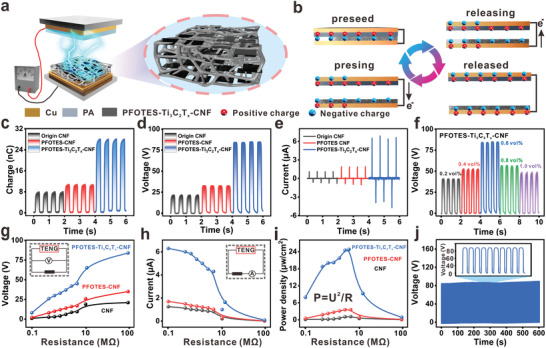
Triboelectric properties of cellulosic triboelectric materials. a) Schematic structure of PFOTES‐Ti_3_C_2_T*_x_*‐CNF TENG. b) PFOTES‐Ti_3_C_2_T*_x_*‐CNF TENG working principle. c) Comparison of the output charges of the three films. d) Comparison of the output voltage of three films. e) Comparison of the output current of three films. f) Comparison of the output voltage of different Ti_3_C_2_T*_x_* content films. g) Comparison of load the output voltage of three films. h) Comparison of the load output current of three films. i) Comparison of the load output power density of three films. j) PFOTES‐Ti_3_C_2_T*_x_*‐CNF TENG the output stability test.

The schematic diagram of TENG composed of nylon and PFOTES‐Ti_3_C_2_T*_x_*‐CNF films as positive and negative triboelectric materials is shown in **Figure** **4**a. Inside the negative triboelectric material is a layered structure consisting of cellulose and Ti_3_C_2_T*_x_* sheets connected. The operating principle is shown in Figure 4b, where the electrical output is achieved by contact initiation and electrostatic induction.

To evaluate the effects of PFOTES modification and Ti_3_C_2_T*_x_* incorporation on the triboelectric polarity of CNF films, pure CNF films, PFOTES‐CNF films, and PFOTES‐Ti_3_C_2_T*_x_*‐CNF films were used as negative triboelectric materials, respectively. Those films with a contact area of 3 × 3 cm^2^ and a thickness of 70 µm. During the electrical measurement, the acceleration (0.2 m s^−2^), ambient humidity 45%RH, and frequency (2 Hz) of the contact‐separation motion were maintained at these values by a linear motor for all the tests. The output electrical properties of all three showed an increasing trend at 2 Hz, and the charge, voltage, and current of the film with the layered structure were increased by 325%, 386%, and 494%, respectively, compared to the pure CNF film (Figure 4c–e). It indicates that both the modification of PFOTES and the layered structure of complex‐containing Ti_3_C_2_T*_x_* contribute significantly to the electrical property output of TENG. It is due to hydrogen bonds between the Ti_3_C_2_T*_x_* surface atoms (F, OH) in the layered structure and the —OH in the cellulose chain. The layered structure increases the surface area of the sheet. It increases the active sites and microcapacitor network, which drives the charge storage capacity of the microcapacitor network, thus contributing to the improved output performance of the TENG.^[^ ^31^ ^]^ The triboelectric properties of PFOTES‐Ti_3_C_2_T*_x_*‐CNF films at different Ti_3_C_2_T*_x_* contents are shown in Figure 4f. The films have the maximum output at the Ti_3_C_2_T*_x_* range of 0.6 vol%. The layered structure is complete at this content, while too little Ti_3_C_2_T*_x_* is incomplete or too much will lead to self‐stacking of Ti_3_C_2_T*_x_* nanosheets, which will eventually affect their output performance.

In addition, the TENG consisting of three films was connected to resistors of different resistance values (10^0^–10^8^ Ω), and the data showed that the current decreased with the increasing resistance, while the output voltage increased with increasing resistance (Figure 4g,h). The three triboelectric materials reached voltages of 21, 35, and 84 V at a high resistance of 9 × 10^9^ Ω, respectively. The instantaneous output power (*P* = *U* ^2^/*R*) of the three films at external load was calculated, and the results showed that the peak values of CNF film, PFOTES‐CNF film, and PFOTES‐Ti_3_C_2_T*_x_*‐CNF film were 1, 3.5, and 25 µW cm^−2^, respectively, at a resistance of 9 × 10^6^ Ω (Figure 4i). The composite film has good output stability, and the film still has stable electrical output performance after 1200 cycles of contact (Figure 4j).

### 2.4 Cellulosic Triboelectric Materials for Ammonia Sensing

**Figure 5:**
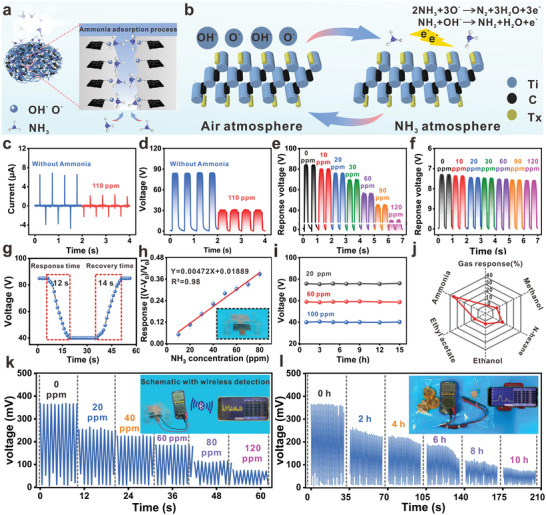
Cellulosic triboelectric materials for ammonia sensing. a) Schematic diagram of NH_3_ adsorption by layered structure. b) Schematic diagram of NH_3_ sensing mechanism. c) Current output graph with and without NH_3_. d) Voltage output graph with and without NH_3_. e) Voltage output plots for exposure to different concentrations of NH_3_. f) Effect of NH_3_ on the electrical properties of nylon film output. g) Real‐time continuous response/recovery process to 100 ppm NH_3_. h) Response fitting curves of TENG at different NH_3_ concentrations. i) TENG long‐term stability test at 20, 60, and 100 ppm NH_3_ for 15 h. j) Selectivity of TENG when exposed to 100 ppm of different interfering gases. k) Wireless sensing voltage signals from exposure to different concentrations of NH_3_. l) Wireless sensing voltage signals at different times of food spoilage.

To evaluate the gas sensing potential of gas‐sensitive CNF‐based triboelectric materials, they were tested as negative triboelectric materials for TENG in different concentrations of NH_3_. The picture of the experimental instrument is shown in Figure S6 in the Supporting Information. The micro/nanoscale interfacially enhanced nanosheet units on the material surface and in the layered structure and the interconnections between them provide abundant active sites for NH_3_, which facilitate the adsorption and desorption of the target gas (**Figure** **5**a). At the same time, NH_3_, as a polar molecule, tends to lose electrons. In the electrostatic field caused by triboelectric electricity, it tends to attach to negative triboelectric materials that can easily gain electrons due to electrostatic adsorption. The sensing mechanism of Ti_3_C_2_T*_x_* for NH_3_ mainly involves the absorption of the sensing gas by defects and functional groups.^[^ ^32^ ^]^ Specifically, gas molecules are adsorbed on the active sites of Ti_3_C_2_T*_x_* nanosheets mainly through the dispersion force between polarized gas molecules and partially charged functional groups or defects. Figure 5b demonstrates that when Ti_3_C_2_T*_x_* is exposed to the NH_3_ atmosphere, NH_3_ molecules can bind to the surface functional groups of Ti_3_C_2_T*_x_*, such as —O and —OH. This process can be explained by Equations (1) and (2)^[^ ^20^ ^]^

(1) $$2{\mathrm{NH}}_{3}+3O^{-}\to N_{2}+3H_{2}O+3e^{-}$$

(2) $${\mathrm{NH}}_{3}+{\mathrm{OH}}^{-}\;\to {\mathrm{NH}}_{2}+H_{2}O+e^{-}$$

The output performance of TENG was evaluated in the presence of NH_3_, as shown in Figure 5c,d. The open‐circuit voltage of TENG decreases at the NH_3_ concentration of 110 ppm compared to that in air. The short‐circuit current value also offers the same trend (declining importance). This is due to the adsorption of NH_3_ molecules on the active sites of Ti_3_C_2_T*_x_* nanosheets, which reduces the binding of PFOTES‐CNF to the active sites and thus leads to a reduction in the microcapacitor network, prompting a decrease in the charge storage capacity of the microcapacitor network. To investigate the sensitivity of TENG for NH_3_, the open‐circuit voltage response was measured at different NH_3_ concentrations, and the results are shown in Figure 5e. The open‐circuit voltage of TENG decreases with increasing NH_3_ concentration. The voltage of TENG decreases from 84 to 25 V as the NH_3_ concentration increases from 0 to 120 ppm. Also, the effect of NH_3_ on the positive nylon film should be considered. As shown in Figure 5f, the output performance of TENG was assembled with pure CNF and nylon, and the nylon film was subjected to different NH_3_ concentration levels with almost no change. Response/recovery time plays a vital role in practical gas sensing applications. Figure 5g shows the TENG's dynamic response/recovery transients to 100 ppm NH_3_. It can be found that the response/recovery time of TENG is 12 and 14 s. The PFOTES‐Ti_3_C_2_T*_x_*‐CNF used in this work showed superior responsiveness as an ammonia‐sensitive triboelectric material compared with other sensing mechanisms NH_3_ sensors (Table S2, Supporting Information).

The sensitivity of the gas‐sensitive CNF‐based triboelectric material is defined as the slope of the voltage change plot (|*V*_0_ − *V*|/*V*_0_) versus NH_3_ concentration, where *V*_0_ and *V* denote the initial voltage in the absence of NH_3_ and the voltage in the presence of NH_3_, respectively. The response value of TENG (*Y*) as a function of NH_3_ concentration (*X*) is shown in Figure 5h, where the most suitable fit is described by the following linearity: *Y* = 0.00472*X* + 0.01889. TENG exhibits good linearity as the NH_3_ sensor. In practical applications, the long‐term stability of the gas sensitivity is the criterion of the sensor. As shown in Figure 5i, the TENG showed good stability at NH_3_ concentrations of 20, 60, and 100 ppm during the 15 h test (sensitivity response *V*_air_ */V*_gas_ = 2.1 at 100 ppm). In addition, the stability and long‐term performance of the sensor were examined by cycling the test 1900 times at 100 ppm ammonia. The stability response of the sensor was good within the first 420 s (840 cycles) and its sensitivity gradually decreased after more than 840 cycles. This is due to the limited ammonia‐sensitive functional groups on the surface of Ti_3_C_2_T*_x_* as the cycle time and the number of cycles increase, the reaction of the gas with the surface of the frictional electric material gradually decreases, and eventually, the sensitivity of the sensor should gradually decrease with the surface reaction with the gas (Figure S7, Supporting Information).^[^ ^33^ ^]^ The response values shown in Figure 5j indicate that the PFOTES‐Ti_3_C_2_T*_x_*‐CNF has great sensitivity to ammonia gas at the same gas concentrations, which is considered for the formation of more adsorption sites on the PFOTES‐Ti_3_C_2_T*_x_*‐CNF film.^[^ ^34^ ^]^ The 2D structure of Ti_3_C_2_T*_x_* gives it a higher specific surface area and can adsorb many end‐joining groups on the surface.^[^ ^35^ ^]^ In the structure of Ti_3_C_2_T*_x_*, the surface functional groups provide many defects that provide a more active state for gas adsorption, and the layered structure provides an efficient electron transport channel, both of which are favorable for gas detection.^[^ ^28^ ^]^ The PFOTES‐CNF and PFOTES‐Ti_3_C_2_T*_x_*‐CNF films were characterized by an automated specific surface area and porosity analyzer, and it was found that the pore size of PFOTES‐Ti_3_C_2_T*_x_*‐CNF film was larger than that of PFOTES‐CNF film, which was mainly due to the effect of the layered structure and the presence of Ti_3_C_2_T*_x_* (Figure S8, Supporting Information). The lamellar structure gave the material a larger pore size providing a more active state for gas adsorption and also effectively improved the adsorption performance of PFOTES‐Ti_3_C_2_T*_x_*‐CNF films. Meanwhile, Ti_3_C_2_T*_x_* has very good selectivity for ammonia. It is attributed to the better adsorption capacity of Ti_3_C_2_T*_x_* for ammonia.^[^ ^36^ ^]^ Therefore, the selectivity of the gas‐sensitive material can also reflect the strength of the adsorption capacity of the gas‐sensitive material. Furthermore, the gas response characteristics of other Ti_3_C_2_T*_x_*‐based ammonia gas sensors reported in the literature are summarized and compared in Table S1 in the Supporting Information. It shows that the response of this work has obvious advantages compared with the other Ti_3_C_2_T*_x_*‐based ammonia gas sensors in terms of the response and response speeds. This reflects that PFOTES‐ Ti_3_C_2_T*_x_*‐CNF film has good gas adsorption properties.

To understand the gas sensing performance of PFOTES‐Ti_3_C_2_T*_x_*‐CNF films in more detail, the overlapping electron cloud (OEC) model is used to explain the electron transfer in the contact electrification phenomenon of PFOTES‐Ti_3_C_2_T*_x_*‐CNF.^[^ ^37^ ^]^ The electron cloud and potential good models are presented as universal features of all materials.^[^ ^38^ ^]^ In the OEC model, a shallowly bounded electron could hop from one atom to the other if the interatomic distance is shorter than the normal bonding length between the two, owing to the lowered potential barrier between the two.^[^ ^39^ ^]^ We fully discuss the changes of potential barriers between atoms on the surface of triboelectric materials before and after the action of ammonia, and the effect of the electric field on electron transfer when the electron clouds overlap through the ECO model. (For details, see Note S1 in Supporting Information).

In addition, the present work provides a more in‐depth analysis of the change of the potential difference between two triboelectric materials after ammonia adsorption based on electron transfer. The potential difference between the two electrodes of the friction nanogenerator is composed of two main parts. One part comes from the polarized frictional charges, which contribute to the voltage as *V*_oc_(*x*) and are related to the separation distance. Besides, the transferred charge Q will also contribute to the potential difference between the two electrodes. The contribution of the transferred charge to the potential difference between the two electrodes is −*Q*/*C*(*x*), where *C* is the capacitance between the two electrodes. Therefore, according to the principle of potential superposition, the total potential difference between the two electrodes is shown as follows.^[^ ^40^ ^]^

(3) $$V=-\frac{1}{C\left(x\right)}Q+V_{\mathrm{oc}}\left(x\right)$$

Equation (3) (called the *V*‐*Q*‐*X* relationship) is the controlling equation for either friction nanogenerator and clearly explains the intrinsic capacitance property.^[^ ^40^ ^]^ The separation of polarized frictional charges will produce a potential difference between the two electrodes. The standard defining equation for a capacitor is shown below.

(4) $$C=\frac{\varepsilon S}{4K\pi x}$$

In the whole process *ε*, *S*, *K*, and *π* are constant values, the only variable is *x*. When the separation distance is certain, we consider that the capacitance is constant, and assume that *C*(*x*) is *C*_1_, then at this time, the potential difference between the two electrodes is^[^ ^40^ ^]^

(5) $$V=-\frac{1}{C_{1}}Q+V_{\mathrm{oc}}\left(x\right)$$

From the above OEC model analysis, it is known that *E*_2_ *D*_2_ < *E*_1_ *D*_2_ before and after ammonia adsorption. The voltage *V*_oc_(*x*) contributed by the polarized frictional charge after ammonia adsorption is smaller than *V*_oc_(*x*) before ammonia adsorption. Then according to Equation (5), the total potential after adsorption is smaller than the total potential before adsorption.

To verify our conjecture, we simulated the total potential difference between the two electrodes at different ammonia concentrations using COSMOL, as shown in Figure S10a–c in the Supporting Information. The voltages were 82, 70, and 40 V at the ammonia concentrations of 0, 40, and 100 ppm, respectively, and the overall trend was also decreasing, which was also consistent with our theoretical analysis and calculation.

Based on this reliable monitoring method, and to better conform to the concept of the Internet of everything and make it more convenient to receive biological signal information, a wireless real‐time sensor system connected to the mobile terminal is designed.^[^ ^41^ ^]^ The system first senses an electrical signal from the NH_3_ sensor of the gas‐sensitive CNF‐based triboelectric material, then transmits it to a digital multimeter and wirelessly transmits the signal to a cell phone application for display through its Bluetooth function (Movie S1, Supporting Information). The experimenter monitored and recorded 10 s cell phone terminal data with TENG at different NH_3_ concentrations to verify this function. The corresponding detection curves are shown in Figure 5k and the trend of voltage signal change on the cell phone application are similar to Figure 5e. it demonstrates the feasibility of a potential application of a gas‐sensitive CNF‐based triboelectric material NH_3_ sensor for wireless real‐time sensing of NH_3_ concentration. In addition, wireless real‐time sensing of ammonia concentration in spoiled food at different periods further validated the strong potential of this gas‐sensitive CNF‐based triboelectric material for NH_3_ monitoring (Figure 5l). Based on the above analysis, gas‐sensitive CNF‐based triboelectric materials have ideal reliability as negative triboelectric materials for self‐powered gas sensing in TENG.

## 3 Conclusion

In summary, a gas‐sensitive CNF‐based triboelectric material with a layered structure was prepared in this work and used for a high‐sensitivity self‐powered ammonia‐sensitive sensor. This self‐powered ammonia sensing sensor has a fast response/recovery (12/14 s), high sensitivity response (*V*_air_/*V*_gas_ = 2.1), high selectivity response (37.6%), and low detection limit (10 ppm) for 100 ppm ammonia gas. In addition, the ammonia‐sensitive sensor can accurately identify NH_3_ concentration changes in the range of 10–120 ppm and wirelessly transmit the signal to the user interface, providing online real‐time monitoring of NH_3_ in the environment. This lays a solid foundation for developing and applying high‐performance gas‐sensitive materials and shows excellent potential and application prospects for scalable gas sensing fields.

## 4 Experimental Section

### Materials

Bleached sugarcane slurry plates were provided by your sugar group. Ti_3_C_2_T*_x_* solution was purchased from Aladdin Reagent Network, concentration 0.5 g/50 mL, ionic size 400 mesh. Triethoxy1H,1H,2H,2H‐tridecafluoro‐*n*‐octylsilane (PFOTES) and ethanol (C_2_H_5_OH) were purchased from Aladdin Chemicals. All samples were of analytical purity grade. Deionized water (DI) was applied in all experimental procedures.

### Chemical Modification of CNF Surface

The PFOTES‐CNF films were prepared according to Nie's method.^[^ ^26^ ^]^ The ethanol to water ratio of 8:2 by volume was used as the hydrolysis solution. The silane hydrolysis product was obtained by adding 1 wt% of PFOTES to the alcohol/water mixture and stirring for 2 h at room temperature. Then, 0.5 g of CNF (dry weight) was added to the solution. After sufficient stirring, the suspension was subjected to ultrasonic treatment at 800 W for 5 min, and finally, the suspension was placed in a constant temperature water bath at 80 °C for 4 h.

### Preparation of PFOTES‐Ti_3_C_2_T*_x_*‐CNF Composite Films

PFOTES‐Ti_3_C_2_T*_x_*‐CNF composite films with a thickness of 70 µm were prepared by intermittent vacuum filtration. The modified PFOTES‐CNF was placed in a 250 mL beaker, 0.6 vol% Ti_3_C_2_T*_x_* solutions were added, and then deionized water was added to dilute the concentration of the sample. The suspension was sonicated for 30 min and stirred at room temperature for 1 h. The well‐dispersed CNF suspension was poured into a G5 sand core funnel equipped with filter paper and a polytetrafluoroethylene (PTFE) membrane. After five intervals of vacuum filtration, the wet CNFs membrane was removed and dried at 60 °C for 20 min using a paper machine. Finally, the dried films were cured at 120 °C to obtain CNF and PFOTES‐Ti_3_C_2_T*_x_*‐CNF films.

### Assembly of PFOTES‐ Ti_3_C_2_T*_x_* ‐CNF‐Based TENG

Nylon and PFOTES‐Ti_3_C_2_T*_x_*‐CNF composite films were used as two pairs of opposite triboelectric materials. First, two layers of 3 cm × 3 cm and 1 mm thick acrylic sheets were cut as the substrate layer. The PFOTES‐Ti_3_C_2_T*_x_*‐CNF composite film and nylon film were cut into 3 cm × 3 cm and then peeled from the aluminum foil. Paste the two triboelectric layers onto the copper foil separately and put a 1 mm thick acrylic sheet (double‐sided adhesive) on the back. A 6 mm thick sponge is attached to the copper foil on either side. Then, electrodes were connected with wires from the copper foil, and finally, electrical measurements were performed by external wiring connections.

### Characterization

The crystal structure of PFOTES‐Ti_3_C_2_T*_x_*‐CNF films was analyzed by XRD (MiniFlex600) using Cu K*α* radiation (*λ* = 1.54 Å). The chemical structure was measured by attenuated total reflectance (ATR)‐FTIR (TENSOR II, Brook technology, Germany) with a test resolution of 0.4 cm^−1^ and a sample test wavelength range of 400–4000 cm^−1^. The chemical composition of PFOTES‐Ti_3_C_2_T*_x_*‐CNF films was analyzed by K‐Alpha XPS (Thermo Fisher, Thermo Scientific NEXSA). The test voltage was 15 kV, and the current was 15 mA. The roughness of the samples was measured at room temperature using an AFM system (Hitachi, AFM5100N, Japan). Tensile stress–strain experiments and tensile self‐healing were examined using a universal electronic material testing machine (3367, Istron, USA). The sample was stretched over an area of 20 × 10 × 2 mm^3^. The output current, voltage, and transferred charge were measured at room temperature using an electrostatic meter (Keithley 6514, USA) and an acquisition card (NI‐USB6259, USA). All electrical performance measurements were performed at room temperature with a relative humidity of 45%RH.

## Conflict of Interest

The authors declare no conflict of interest.

## Supporting information

Supporting Information

Supplemental Movie 1

## Data Availability

The data that support the findings of this study are available from the corresponding author upon reasonable request.
