# Supplementary material for: Gas‐Sensitive Cellulosic Triboelectric Materials for Self‐Powered Ammonia Sensing
Source: Adv Sci (Weinh). 2022 Aug 26;9(30):2203428. doi: 10.1002/advs.202203428 (PMC9596830; doi:10.1002/advs.202203428)
Supplement: Supplementary file 1 — Supporting Information [file ADVS-9-2203428-s001.pdf]

ADVANCED SCIENCE
Open Access

Supporting Information

for *Adv. Sci.*, DOI 10.1002/advs.202203428

Gas-Sensitive Cellulosic Triboelectric Materials for Self-Powered Ammonia Sensing

*Wanglin Zhang, Jiamin Zhao, Chenchen Cai, Ying Qin, Xiangjiang Meng, Yanhua Liu and Shuangxi Nie**

Supporting Information

Gas-Sensitive Cellulosic Triboelectric Materials for Self-Powered Ammonia Sensing

*Wanglin Zhang, Jiamin Zhao, Chenchen Cai, Ying Qin, Xiangjiang Meng, Yanhua Liu, Shuangxi Nie**

W. Zhang, J. Zhao, C. Cai, Y. Qin, X. Meng, Y. Liu, Prof. S. Nie.
School of Light Industry and Food Engineering, Guangxi University, Nanning, 530004, PR China.
E-mail: nieshuangxi@gxu.edu.cn

Note: One

On the other hand, PFOTES-$Ti_3C_2T_x$-CNF films are used as gas-sensitive triboelectric materials, and we also fully discuss the variation of gas sensing properties from the triboelectric point of view. The usual overlapping electron cloud (OEC) model is used to explain the electron transfer in all types of Contact electrification (CE) phenomena in general materials, including insulators. [1] This is because CE can occur in all materials and under any conditions.[2] In the OEC model, a shallowly bounded electron could hop from one atom to the other if the interatomic distance is shorter than the normal bonding length between the two, owing to the lowered potential barrier between the two. [2] In this work, PFOTES-$Ti_3C_2T_x$-CNF films and nylon were used as positive and negative frictional electric materials, respectively. As shown in Fig. S9a, when the separation between two atoms is large, the potential barrier between the atoms still exists and the electrons of the atoms cannot get enough energy to pass through the barrier. Therefore, there is no electron transfer between the samples. In contrast, when subjected to external stimuli, in the absence of ammonia, the electric field affects the electron transfer if the electron clouds overlap. As shown in Figure S9b, when the electron clouds overlap, electron transfer between PFOTES-$Ti_3C_2T_x$-CNF films and nylon films, respectively, occurs, and the potential barrier decreases at this time. Moreover, a potential difference in E_1D_2 is generated between the two atoms. the gradient of the potential field between the two atoms depends on the strength of the interfacial electric field. When in the atmosphere of ammonia, ammonia can have a series of reactions with the surface of PFOTES-$Ti_3C_2T_x$-CNF films, which will reduce its surface polarity and the reaction products also weaken the ability of its surface to gain electrons. As shown in Figure S9c, when the electron clouds of atoms overlap under the effect of ammonia, the transferred electrons are affected and the final surface charge number will be smaller than the surface charge number in the absence of ammonia. The final potential difference is $E_2D_2 < E_1D_2$. If the electric field is not strong enough, electrons cannot escape from the potential well-formed by the atoms due to the potential barrier. As the electric field strength increases, the gradient of the potential field at the interface also increases. The charge barrier that traps the electrons in the potential well remains as the potential field gradient increases, but the thickness of the barrier decreases. As shown in Figure S9d, if the electric field is stronger than the threshold, electron tunneling occurs because the potential barrier is too thin to trap the electrons (red potential curve) [2].

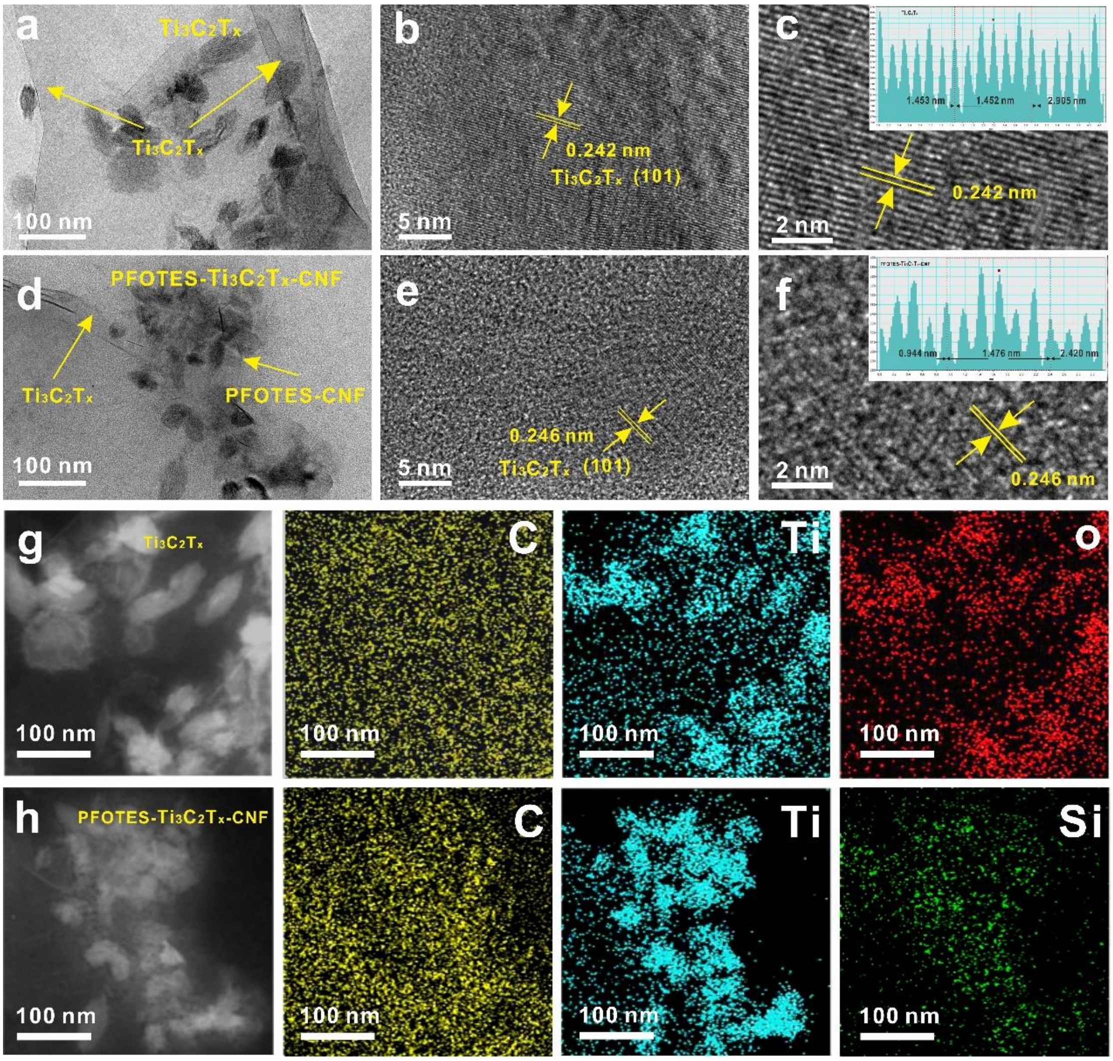

Figure S1. HRTEM images and corresponding mapping results of $Ti_3C_2T_x$ and PFOTES-$Ti_3C_2T_x$-CNF. a) HRTEM image of the $Ti_3C_2T_x$. b, c) The HRTEM image of the edge of the sheet in $Ti_3C_2T_x$. d) HRTEM image of the $Ti_3C_2T_x$. e, f) The HRTEM image of the edge of the sheet in $Ti_3C_2T_x$. g) Corresponding mapping result of the $Ti_3C_2T_x$. h) Corresponding mapping result of the PFOTES-$Ti_3C_2T_x$-CNF.

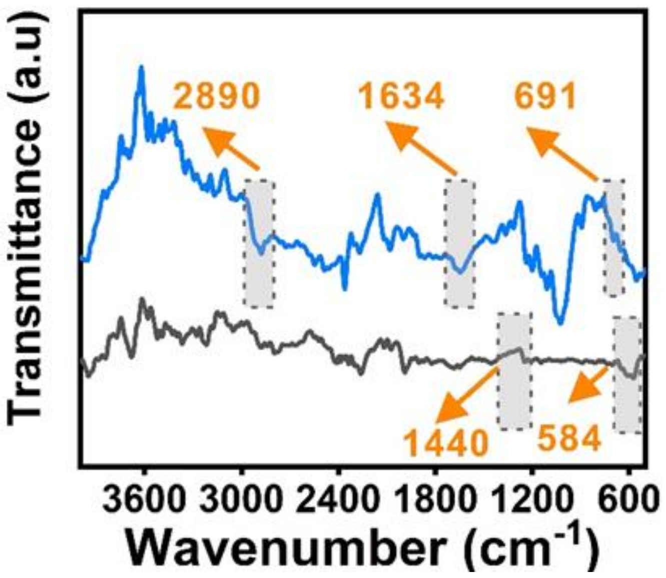

Figure S2. FTIR spectra of gas-sensitive cellulosic triboelectric materials.

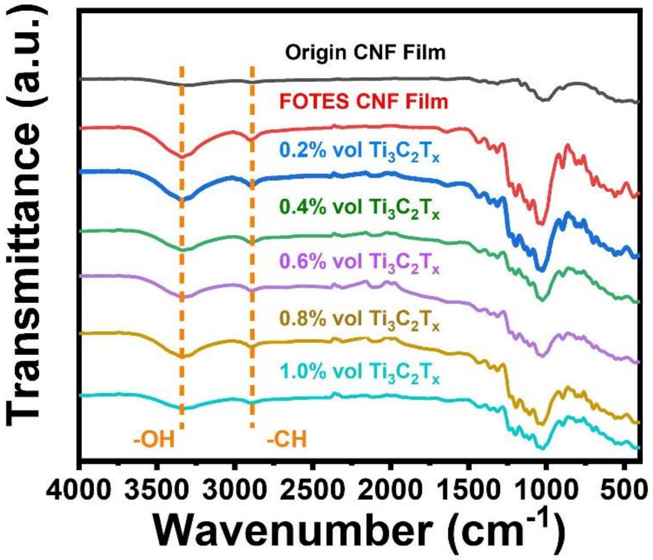

Figure S3. FTIR characterization of PFOTES-$Ti_3C_2T_x$-CNF film with different contents of $Ti_3C_2T_x$.

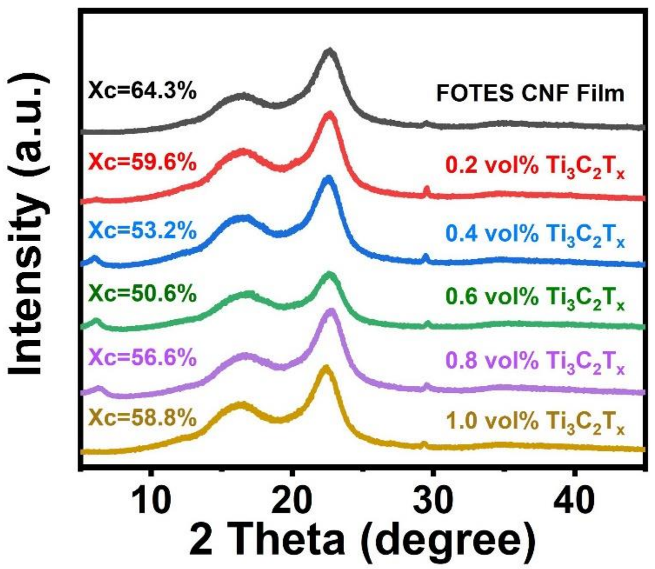

Figure S4. Infrared characterization of PFOTES-Ti_3C_2Tx-CNF film with different contents of Ti_3C_2Tx.

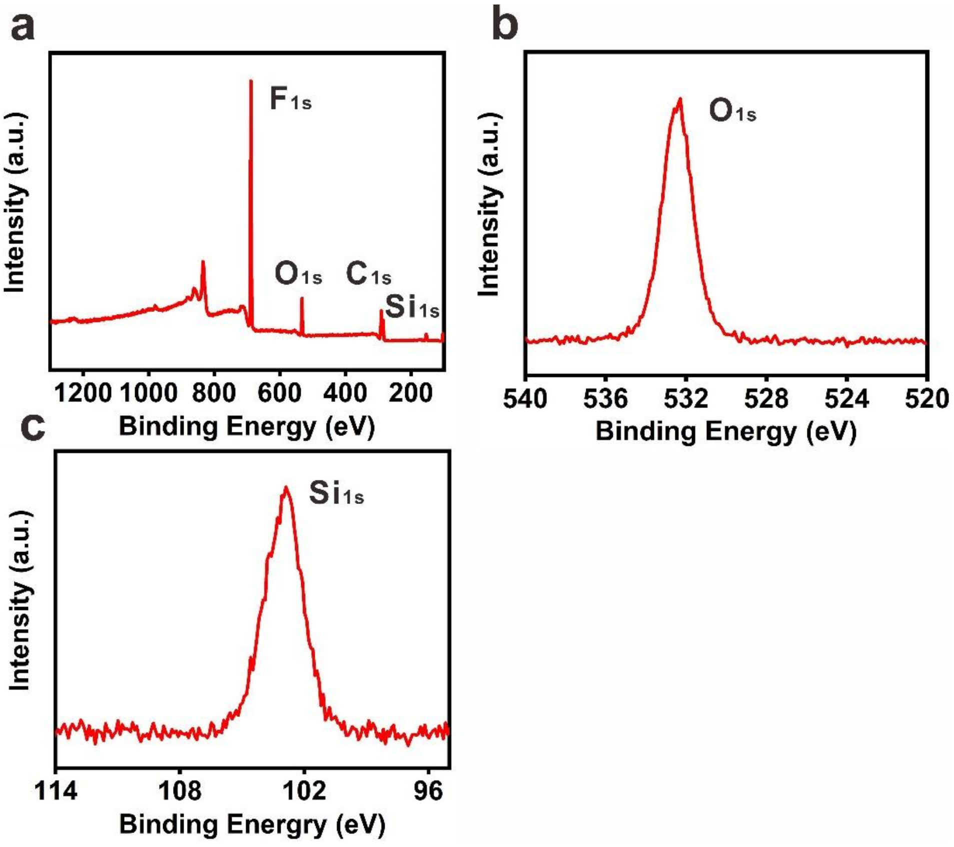

Figure S5. a) Survey Spectrum. b) O1s of PFOTES-Ti_3C_2Tx-CNF composite film. c) Si1s of PFOTES-Ti_3C_2Tx-CNF composite film.

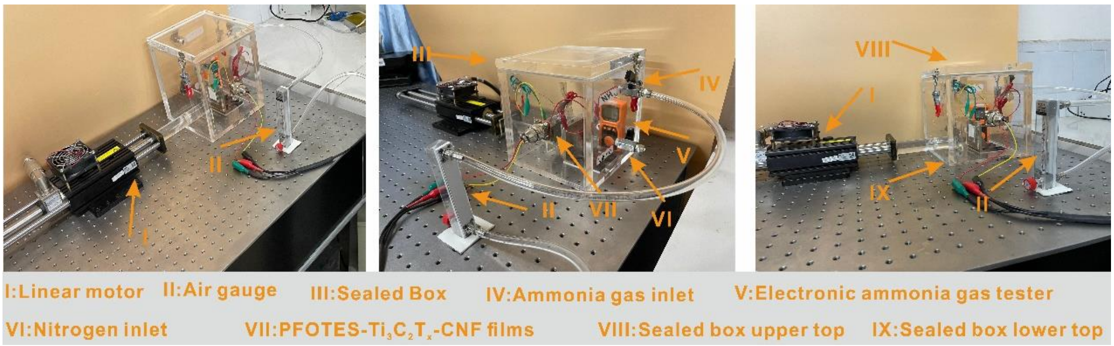

Figure S6. Pictures of instruments for ammonia sensing.

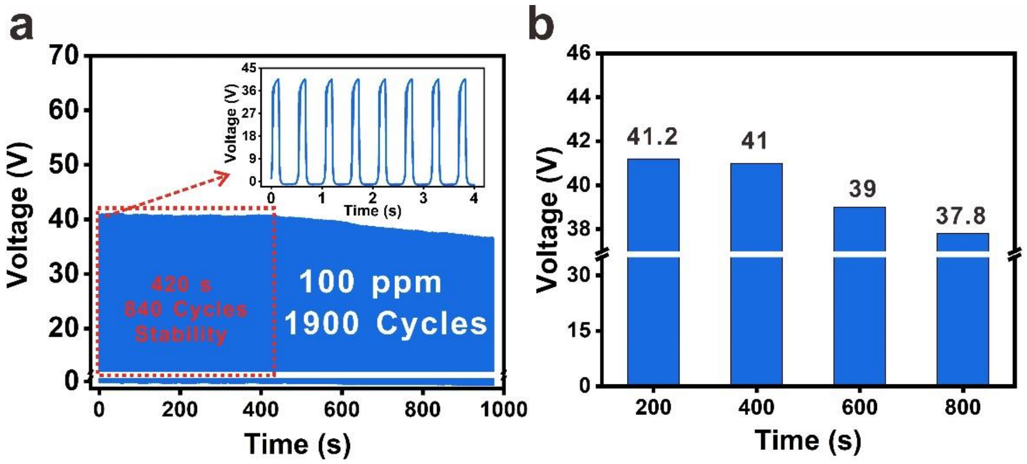

Figure S7. a) The stability test of triboelectric materials under ammonia gas 100 ppm. b) Output voltage for different periods

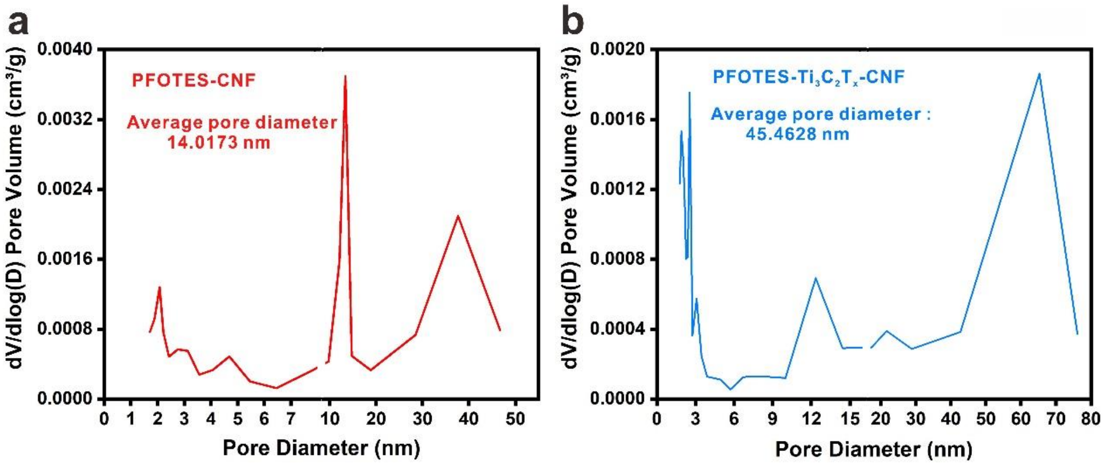

Figure S8. Average pore size of different films. a) PFOTES-CNF film. b) PFOTES-$Ti_3C_2T_x$-CNF film.

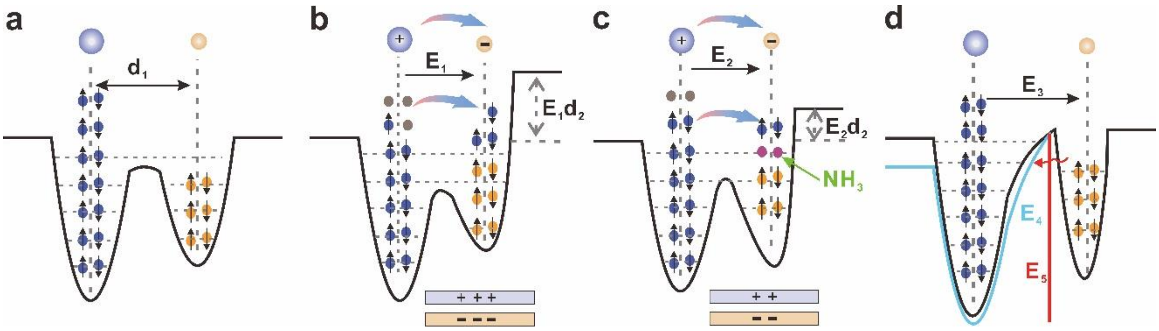

Figure S9. The overlapped electron-cloud model (Wang transition) on the CE. a) Electron clouds do not overlap. b) Electron cloud model in the absence of ammonia. c) Electron cloud model in the presence of ammonia. e) out of the repulsive region with the different electric fields at the interface and the tunneling of electrons (E2 < E3 < E4).

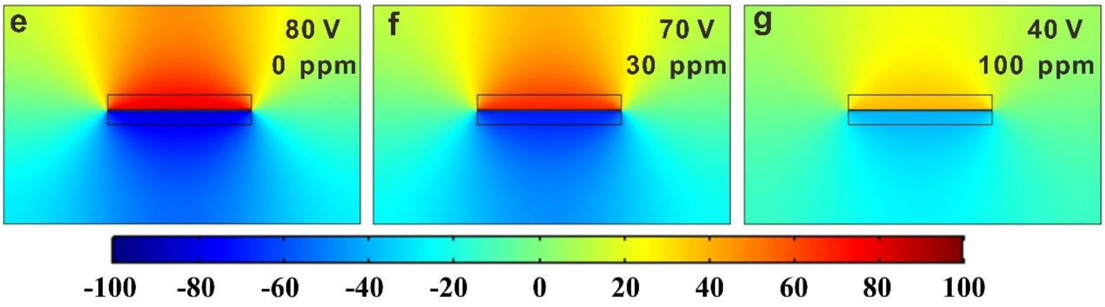

Figure S10. COSMOL simulation of the total potential difference between two electrodes at different ammonia concentrations. a) 0ppm.b) 30 ppm. c) 100 ppm.

Table S1. Comparison of ammonia sensing properties of $Ti_3C_2T_x$ based gas sensors reported in the literature.

$Ti_3C_2T_x$ based nanomaterials	Concentration of ammonia	Response	References
PFOTES-$Ti_3C_2T_x$-CNF	100 ppm	37.6%	This work
Ti_3C_2	500 ppm	6.2%	[3]
$Ti_3C_2T_x$	100 ppm	0.8%	[4]
$Ti_3C_2T_x$	100 ppm	21%	[5]

$Ti_3C_2T_x/TiO_2$	10 ppm	3.1%	[6]
Alkalized MXene	100 ppm	28.87%	[7]
PANI/$Ti_3C_2T_x$	10 ppm	1.65%	[8]
V_2CT_x	100 ppm	1.66%	[9]

Table S2. Comparison of the NH_3 sensor based on other sensing mechanisms.

Sensor materials	Response time (s)	Recovery time (s)	References
PFOTES-$Ti_3C_2T_x$-CNF	11	14	This work
Perovskite quantum dots	10	30	[10]
Berlin green	88	42	[11]
PSS/MXene	116	40	[12]
Carbon nanotube doped polypyrrole	90	450	[13]
MXene/Metal-Organic Framework	45	29	[14]
ZnO-PANI	109	233	[15]
PANI-MWCNTs	120	137	[16]
Polyaniline nanowires	40	/	[17]

References

[1] A. C. Wang, B. Zhang, C. Xu, H. Zou, Z. Lin, Z. L. Wang, Adv. Funct. Mater. 2020, 30 (12), 1909384.

[2] S. Lin, C. Xu, L. Xu, Z. L. Wang, Adv. Funct. Mater. 2020, 30 (11), 1909724.

[3] M. Wu, M. He, Q. Hu, Q. Wu, G. Sun, L. Xie, Z. Zhang, Z. Zhu, A. Zhou, ACS sensors 2019, 4, 2763.

[4] S. J. Kim, H.-J. Koh, C. E. Ren, O. Kwon, K. Maleski, S.-Y. Cho, B. Anasori, C.-K. Kim, Y.-K. Choi, J. Kim, ACS Nano 2018, 12, 986.

[5] E. Lee, A. VahidMohammadi, B. C. Prorok, Y. S. Yoon, M. Beidaghi, D.-J. Kim, ACS applied materials & interfaces 2017, 9, 37184.

[6] H. Tai, Z. Duan, Z. He, X. Li, J. Xu, B. Liu, Y. Jiang, Sensors and Actuators B: Chemical 2019, 298, 126874.

[7] Z. Yang, A. Liu, C. Wang, F. Liu, J. He, S. Li, J. Wang, R. You, X. Yan, P. Sun, ACS sensors 2019, 4, 1261.

[8] X. Li, J. Xu, Y. Jiang, Z. He, B. Liu, H. Xie, H. Li, Z. Li, Y. Wang, H. Tai, Sensors and Actuators B: Chemical 2020, 316, 128144.

[9] E. Lee, A. VahidMohammadi, Y. S. Yoon, M. Beidaghi, D.-J. Kim, ACS sensors 2019, 4, 1603.

[10] H. Huang, M. Hao, Y. Song, S. Dang, X. Liu, Q. Dong, Small 2020, 16 (6), 1904462.

[11] T. Yang, L. Gao, W. Wang, J. Kang, G. Zhao, D. Li, W. Chen, H. Zhang, Nano-micro letters 2021, 13 (1), 1.

[12] L. Jin, C. Wu, K. Wei, L. He, H. Gao, H. Zhang, K. Zhang, A. M. Asiri, K. A. Alamry, L. Yang, ACS Applied Nano Materials 2020, 3 (12), 12071

[13] J. Chang, C. Zhu, Z. Wang, Y. Wang, C. Li, Q. Hu, R. Xu, T. Du, M. Xu, L. Feng, Nano Energy 2022, 98, 107271.

[14] D. Wang, D. Zhang, Y. Yang, Q. Mi, J. Zhang, L. Yu, ACS Nano 2021, 15 (2), 2911.

[15] S. Wang, H. Tai, B. Liu, Z. Duan, Z. Yuan, H. Pan, Y. Su, G. Xie, X. Du, Y. Jiang, Nano Energy 2019, 58, 312.

[16] S. Wang, G. Xie, H. Tai, Y. Su, B. Yang, Q. Zhang, X. Du, Y. Jiang, Nano Energy 2018, 51, 231.

[17] Y. Liu, Y. Zheng, Z. Wu, L. Zhang, W. Sun, T. Li, D. Wang, F. Zhou, Nano Energy 2021, 79, 105422.
